# Growth monitoring and mortality risk in low birthweight infants: a birth cohort study in Burkina Faso

**DOI:** 10.12688/gatesopenres.13231.2

**Published:** 2021-08-20

**Authors:** Martha Mwangome, Moses Ngari, Paluku Bahwere, Patrick Kabore, Marie McGrath, James A. Berkley

**Affiliations:** 1 The Childhood Acute Illness and Nutrition Network,, CHAIN, Nairobi, P.O Box 43640-00100,, Kenya; 2 Clinical, KEMRI/Wellcome Trust Research Program, Kilifi, Kilifi, 80108, Kenya; 3 School of Public Health, Center of Research in Epidemiology Biostatistics and Clinical Research,, Université Libre de Bruxelles,, Brussels,, Belgium, Belgium; 4 Valid International, N/A, 35 Leopold Street, Oxford,, Oxford, OX4 1TW,, UK; 5 Africa Regional office,, World Health Organisation,, Brazzaville,, Republic of Congo, Congo; 6 Emergency Nutrition Network, 69 High Street, Marlborough House, Kidlington, Oxfordshire, OX5 2DN, UK; 7 Centre for Clinical Vaccinology & Tropical Medicine, University of Oxford,, Churchill Hospital Old Road,, Headington Oxford, OX3 7LE, UK

**Keywords:** Low birthweight, Infants, Anthropometry, Growth, Mortality

## Abstract

**Background:** Wasting and underweight in infancy is an increasingly recognised problem but consensus on optimum assessment is lacking. In particular, there is uncertainty on how to interpret anthropometry among low birth weight (LBW) infants who may be growing normally. This research aimed to determine growth of infants from birth to two months (around age of vaccination) and the mortality risk of underweight LBW infants compared to normal birth weight (NBW) infants at two and six months age.

**Methods:** A secondary analysis of a birth cohort of 1103 infants in Burkina Faso was conducted. Anthropometry was performed monthly from 0 to 12 months. We assessed associations with mortality using Cox proportional hazards models and assessed discriminatory values using area under receiver operating characteristics curves.

**Results:** Eighty-six (7.8%) children died by age one year, 26/86 (30%) and 51/86 (59%) within two and six months, respectively. At age two months, weight gain since birth did not better discriminate mortality risk than current weight-for-age (P=0.72) or mid-upper arm circumference (P=0.21). In total, 227 (21%) LBW infants had increased risk of mortality: adjusted hazards ratio (aHR) 3.30 (95%CI 2.09 to 4.90). Among infants who were underweight at two and six months, LBW infants (64% and 49%, respectively) were not at reduced risk of death compared to NBW infants (aHR 2.63 (95%CI 0.76 to 9.15) and 2.43 (95%CI 0.74 to 7.98), respectively).

**Conclusion:** Assessing weight gain since birth does not offer advantages over immediate anthropometry for discriminating mortality risk. LBW infants who are later identified as underweight require care to help prevent mortality.

## Abbreviations

LAZ – Length-for-age Z score; LBW – Low birth weight; LMIC -Low- and middle-income countries; MUAC – Mid-upper arm circumference; NBW – Normal birth weight; SAM – Severe acute malnutrition; u6m – Under 6 months; WLZ – Weight-for-length Z score; WAZ – Weight-for-age Z score; MAMI – Management of At risk Mothers and Infants

## Introduction

Infancy is the period of fastest relative growth; on average, a normally growing infant more than doubles their birth weight in the first six months of life :The WHO Child Growth Standards 2006: [Available from: http://www.who.int/childgrowth/standards/en/]. In early infancy, apparent wasting or underweight may occur due to growth faltering and/or as a result of having been born preterm, low birth weight (LBW) or small for gestational age ^1^.

The global prevalence of LBW is 15%, representing more than 20 million infants, 91% of whom are in low and middle-income countries (LMICs) ^2^. In LMICs, birthweight and subsequent anthropometry in infancy and childhood are predictive of both short and longer-term mortality ^3^. Studies report ongoing extra-uterine weight and length restriction and reduced physical strength through to adolescence following LBW compared to normal birth weight (NBW) infants ^4^. The rate of infant growth irrespective of birth weight is influenced by nutritional intake, absorption and assimilation of nutrients, nutrient losses due to infection, other acute or chronic diseases, and genetic or epigenetic predisposition ^5^.

High-quality growth references standards exist for preterm infants from the INTERGROWTH-21 ^st^ study ^6^. However, information on gestational age is often unavailable or unreliable in LMICs, hence WHO growth standards are usually applied to all infants irrespective of birth size. In growth monitoring or nutrition programs, LBW babies may be classified as underweight or wasted whilst having a normal (preterm) growth velocity tracking lower percentiles or ‘catching up’. Anthropometric indicators such as weight-for-length Z score (WLZ) and mid-upper arm circumference (MUAC) identify a large proportion of infants with a history of LBW. For example, in Kenya, 43% of ill hospitalized malnourished infants under six months old had a history of small size at birth ^1^, while in India, LBW was a strong predictor of severe wasting among infants below the age of six months ^7^.

Thus, there is uncertainty among health workers regarding interpretation of anthropometry in LBW infants, with low expectations of growth in LBW infants common and “slow” and potentially “poor” growth in infants (underweight) regarded as acceptable ^8^. Additionally, practitioners target catch up growth of LBW infants to that of peers and assume that mortality risk is resolved among LBW infants who are no longer classified as undernourished ^9^. There are also concerns for potential future health risks associated with accelerated weight gain ^10, 11^. Whether and how to intervene on undernourished LBW infants has implications for health system workload and costs. Consequently, to evaluate risks associated with anthropometry and types of interventions needed, birthweight may need to be considered during anthropometric assessment in infancy. In practice, vaccination at around two months of age is an established opportunity to assess growth and to intervene.

Given this background, we examined data from a birth cohort to compare the discriminatory value for mortality for anthropometry at birth and changes from birth to the following timepoints in infancy: i) at two months of age which is around the time of infant immunisations; and ii) at six months of age. We also investigated overall mortality risk among LBW infants and whether among infants with low anthropometric values measured at two and six months of age, LBW was associated with lower risk of subsequent mortality.

## Methods

### Study site

Data utilized for this secondary analysis was from a birth cohort within Barsalogho Health District, part of the Kaya Health Region in Central North Burkina Faso. It was collected between 1 ^st^ April and 31 ^st^ December of 2004 in four health centers, including Barsalogho, Basma, Dablo, and Foubé. Though old, the dataset is valuable because it contains follow-up data of an untreated infant cohort, which would be difficult to generate at the present time. An untreated cohort offer a more natural experience of growth patterns within the study population. Additional details on the study site can be found in a previous publication ^3^.

### Study population and design

The study cohort recruited pregnant women in their third trimester attending scheduled antenatal care visits. The objective of the original study was to compare survival in infancy of full-term LBW infants to that of full-term NBW infants ^12, 13^. In this secondary analysis, we included data from all live births within the cohort. Follow-up was from birth to 12 months of age through scheduled monthly clinic visits.

### Variables

The main outcome of interest was mortality confirmed through hospital records or burial permits/death certificates. Deaths were included in this analysis if they occurred within the first year of life. Anthropometry was collected monthly from birth up to 12 months of age. Exposures examined were anthropometry at birth and ages two and six months (weight (kg), MUAC (cm) and length (cm)) and demographic factors.

### Data source/measurements

Both the caregiver and infant demographics and anthropometric measurements were collected at birth, usually within two hours for health facility birth and 48 hours for births in the community by a trained community health worker. MUAC in centimetres were measured with a non-stretch measuring tape to the nearest one mm. An electronic scale (Seca 825 Birmingham, UK) was used to measure weight in kilograms. Length in centimetres were measured using an infantometer (Seca 416, Birmingham, UK). Anthropometric z-scores were calculated using WHO (2006) reference WHO; WHO growth standards STATA macro 2011 [Available from: http://www.who.int/childgrowth/software/en/]. Underweight was defined as weight-for-age z-score (WAZ) <-2. LBW was defined as <2.5kg.

### Study size

The parent birth cohort recruited 1103 infants. This secondary analysis included all the 1103 infants. With 1103 infants provide a 7.8% probability of death in one year, a two-sided alpha level of 0.05, the study had power >90% to estimate adjusted hazard ratio of ≥2.25 of LBW associated with death and with power >80% to estimate similar hazard ratio from month two.

Even though the secondary analysis included all the 1103 infants, analyses at different time points used varying number of infants who were alive and in follow-up at the respective time-points.

### Statistical analysis

Infant anthropometric measurements were summarised as means and standard deviation. Maternal age was reported as median and interquartile range. We calculated Medians (IQR) weight increments from birth to month 2 and 6 and compared with the WHO 2009 weight increment standards using Wilcoxon single sign rant test [Available from https://www.who.int/childgrowth/standards/velocity/tr3_velocity_report.pdf]. To estimate hazards ratios for death associated with anthropometry, we used Cox proportional hazards regression adjusted for features presumed to have biological association with anthropometry such as sex, birth weight, prematurity and being a twin. We assessed and found evidence of unobserved heterogeneity between the four sites using likelihood ratio test (P=0.002) in a regression model assessing effects of LBW on one year mortality. To account for this unobserved heterogeneity, we used shared gamma frailty model. We assessed and found no evidence of violation of proportional hazard assumption using the Schoenfeld residuals.

To test the discrimination of mortality risks from month two to twelve months of age by anthropometric measurements at birth, at month two and the change between birth and month two measurements, we estimated the area under receiver operating characteristics curves (AUC). We used the STATA version 15.1 “ *roccomp”* command to test the hypothesis that the AUCs were equal by comparing AUCs from a single time-point (month 2) with the change from birth to month two.

We examined differences in WAZ, and proportion of infants underweight at month two stratified by birth weight and used an independent t-test to test for differences in WAZ between infants born NBW and LBW.

### Ethical considerations

The original birth cohort was approved by the Ministry of Health of Burkina Faso (approval number: 1014) in 2003 in accordance with national procedure. All study participants provided written consent to take part in the original study. All data were anonymized before being shared for this analysis.

## Results

### Cohort characteristics

**Figure 1.**
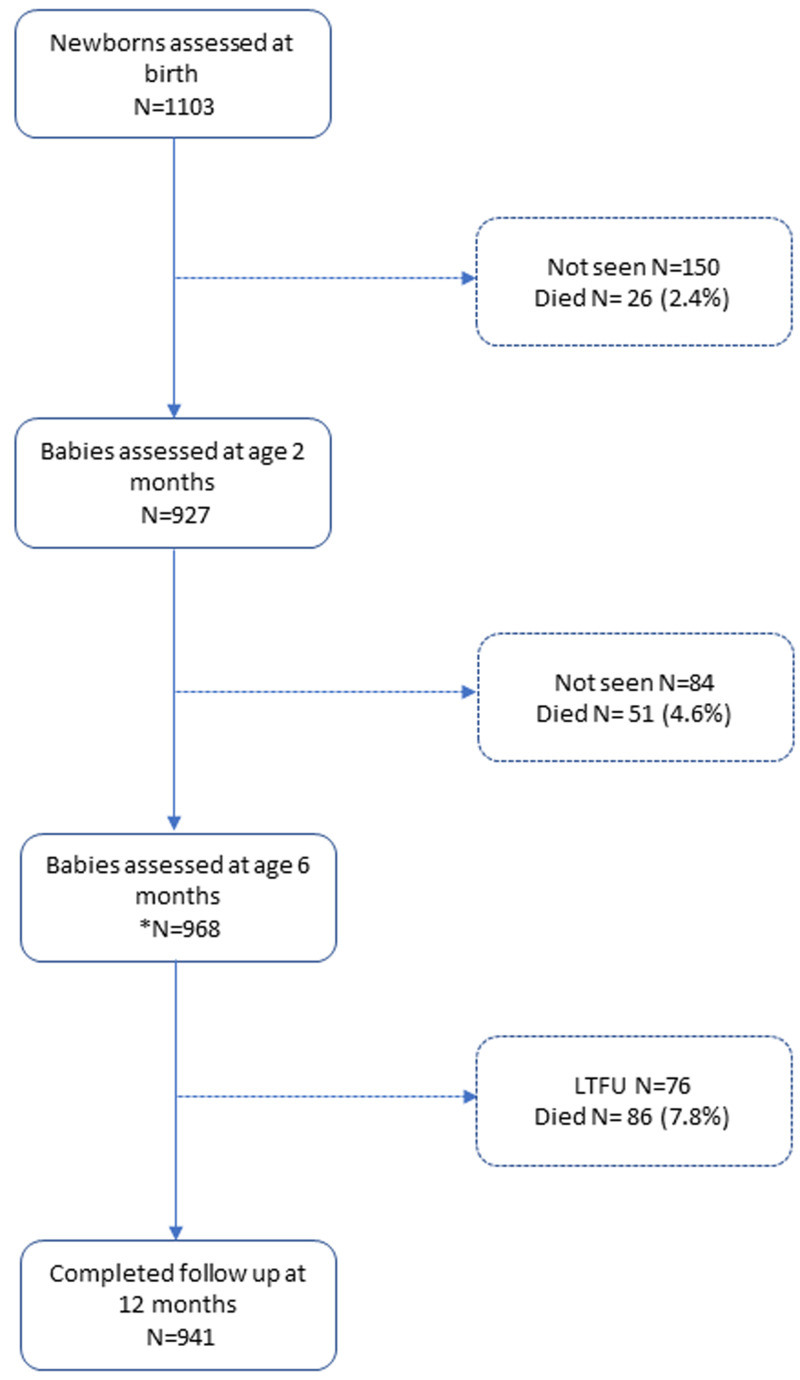
Flow diagram of study participants from recruitment to 12 months follow-up.

The parent birth cohort recruited 1103 infants (( Figure 1), 570 (52%) males and 533 (48%) females. A total 492 (45%) of the infants were born in a health facility, 432 (39%) at home assisted by a community birth attendant and 179 (16%) not assisted by a community birth attendant. The median (IQR) gestation age was 39 (38 to 40) weeks, and 62 (5.6%) were born premature. The mean (sd) birth weight and WAZ were 2.8 (0.5) kg and -1.4 (1.6) Z, respectively ( Table 1). Of the 1103 infants, 227 (21% (95% CI 18 to 23%)) were born LBW. During the one year follow-up, 507 (46%), 777 (70%) and 544 (49%) infants had at least one episode of diarrhoea, fever and cough respectively. Fifty two (4.7%) infants were not exclusively breastfed, while 278 (25%), 773 (70%) were exclusively breastfed for three and six months respectively (Table 1).

**Table 1.** Participants characteristics at birth.

	All infants (N=1,103)	NBW infants (N=876)	LBW infants (N=227)
Demographics			
Sex, N (%)			
Male	570 (52)	477 (54)	93 (41)
Birthplace, N (%)			
Health facility	492 (45)	399 (46)	93 (41)
Home with CBA	432 (39)	349 (40)	83 (37)
Home with no CBA	179 (16)	128 (14)	51 (22)
Recruitment health centre, N (%)			
Basma	416 (38)	323 (37)	93 (41)
CMA	320 (29)	261 (30)	59 (26)
Dablo	286 (26)	227 (26)	59 (26)
Foube	81 (7.3)	65 (7.4)	16 (7.1)
Born premature (gestation age <37 weeks) N (%)	62 (5.6)	14 (1.6)	48 (21)
**Anthropometry**			
Weight (Kg); mean ±sd	2.8 ± 0.5	3.0 ± 0.3	2.2 ± 0.3
MUAC in cm; mean ±sd	10.2 ± 1.1	10.5 ± 0.9	9.2 ± 0.9
Length (cm), mean ±sd	48.9 ± 2.6	49.5 ± 2.1	46.4 ± 2.8
WLZ; mean ±sd	-1.4 ± 1.6	-1.1 ± 1.5	-2.8 ± 1.2
WAZ; mean ±sd	-1.1 ± 1.1	-0.8 ± 0.7	-2.7 ± 0.8
LAZ; mean ±sd	-0.3 ± 1.4	-0.01 ± 1.1	-1.6 ± 1.5
ZHC; mean ±sd	-1.3 ± 1.5	-1.0 ± 1.4	-2.5 ± 1.6
Infant co-morbidity			
Any diarrhea episode	507(46)	396(45)	111(49)
Any fever episode	777 (70)	614 (70)	163 (72)
Any cough episode	544 (49)	424 (48)	120 (53)
Exclusive breastfeeding			
None	52 (4.7)	38 (4.3)	14 (6.2)
First 3 months	278 (25)	226 (26)	52 (23)
Four to six months	773 (70)	612 (70)	161 (71)
**Maternal demographics**			
Mother age; median (IQR) years	25 (20-30)	25 (20-30)	22 (19-30)
Illiterate N (%)	854 (77)	666 (76)	188 (83)
Height (cm); median (IQR)	163 (159–168)	164 (160–168)	163 (157–167)
ANC visits			
None	60 (5.4)	38 (4.3)	22 (9.7)
1 to 3	1024 (93)	822 (94)	202 (89)
≥4	19 (1.7)	16 (1.8)	3 (1.3)
Distance to nearest health facility (kilometres); median (IQR)	7 (2–15)	7 (2–15)	8 (2–15)

CBA-community based assistant, MUAC-mid-upper arm circumference, WLZ-Weight-for-length z-score, WAZ-Weight-for-age z-score, LAZ-Length-for-age z-score, ZHC-Head circumference z-score, sd-Standard deviation, NBW-Normal birth weight (≥2.5kg), LBW-Low birth weight (<2.5kg).

At two months of age, of 927 infants who had anthropometry assessed, 148 (16%) were underweight; 94 (64%) of whom were LBW. At six months of age, of the 968 infants who had anthropometry assessed, 236 (24%) were underweight; 92 (39%) of whom were LBW. Anthropometry by month and LBW is shown in Table 2.

**Table 2.** Monthly weight-for-age z-score and proportion of children underweight stratified by birth weight.

	Birth	Month 1	Month 2	Month 3	Month 4	Month 5	Month 6	Month 7	Month 8	Month 9	Month 10	Month 11	Month 12
All infants													
N	1103	938	927	986	940	916	968	917	917	938	899	887	941
Mean WAZ ±sd	-1.1 ±1.1	-0.7 ±1.3	-0.8 ±1.3	-0.9 ±1.3	-1.0 ±1.3	-1.1 ±1.2	-1.3 ±1.2	-1.4 ±1.2	-1.5 ±1.2	-1.5 ±1.2	-1.6 ±1.2	-1.6 ±1.2	-1.6 ±1.2
WAZ<-2 (%)	185 (17)	134 (14)	148 (16)	159 (16)	171 (18)	176 (19)	236 (24)	278 (30)	304 (33)	304 (32)	310 (34)	298 (34)	307.(33)
Normal birth weight													
N (%) ^#^	876 (79)	745 (79)	740 (80)	786 (80)	756 (80)	739 (81)	780 (81)	741 (81)	739 (81)	762 (81)	730 (81)	724 (82)	762 (81)
Mean WAZ ±sd	-0.7 ±0.7	-0.3 ±1.0	-0.5 ±1.1	-0.6 ±1.1	-0.7 ±1.1	-0.9 ±1.1	-1.1 ±1.1	-1.2 ±1.1	-1.3 ±1.2	-1.4 ±1.2	-1.4 ±1.1	-1.4 ±1.1	-1.4 ±1.1
WAZ<-2 (%)	0	35 (4.7)	54 (7.3)	73 (9.3)	95 (13)	109 (15)	144 (18)	183 (25)	208 (28)	209 (27)	209 (29)	203 (28)	205 (27)
Low birth weight													
N (%) ^#^	227 (21)	193 (21)	187 (20)	200 (20)	184 (20)	177 (19)	188 (19)	176 (19)	178 (19)	176 (19)	169 (19)	163 (18)	179 (19)
Mean WAZ ±sd	-2.7 ±0.8	-2.2 ±1.4	-2.2 ±1.4	-2.0 ±1.5	-2.0 ±1.4	-2.0 ±1.3	-2.2 ±1.3	-2.2 ±1.3	-2.2 ±1.2	-2.3 ±1.2	-2.3 ±1.1	-2.3 ±1.2	-2.3 ±1.2
WAZ<-2 (%)	185 (82)	99 (51)	94 (50)	86 (43)	76 (41)	67 (38)	92 (49)	95 (54)	96 (54)	95 (54)	101 (60)	95 (58)	102 (57)
P-value *	<0.001	<0.001	<0.001	<0.001	<0.001	<0.001	<0.001	<0.001	<0.001	<0.001	<0.001	<0.001	<0.001

WAZ-Weight-for-age z-score, *the P-value is from the comparison of the means of WAZ between infants born LBW and normal birth weight using independent t-test, #-percentage of all infants in that follow-up visit.

During twelve months of follow-up, 86 (7.8%) infants died and 76 (6.9%) were lost to follow up after a median of 154 (IQR 91 to 247) days. The total period of observation was 1015 child/years ( Figure 1). Twenty-six (30%) deaths occurred before two months of age. LBW was associated with an increased risk of death during the first year of life: hazards ratio (HR) 3.3 (95% CI 2.09 to 4.90) and P<0.001.

### Anthropometric changes from birth and their association with mortality

**Figure 2.**
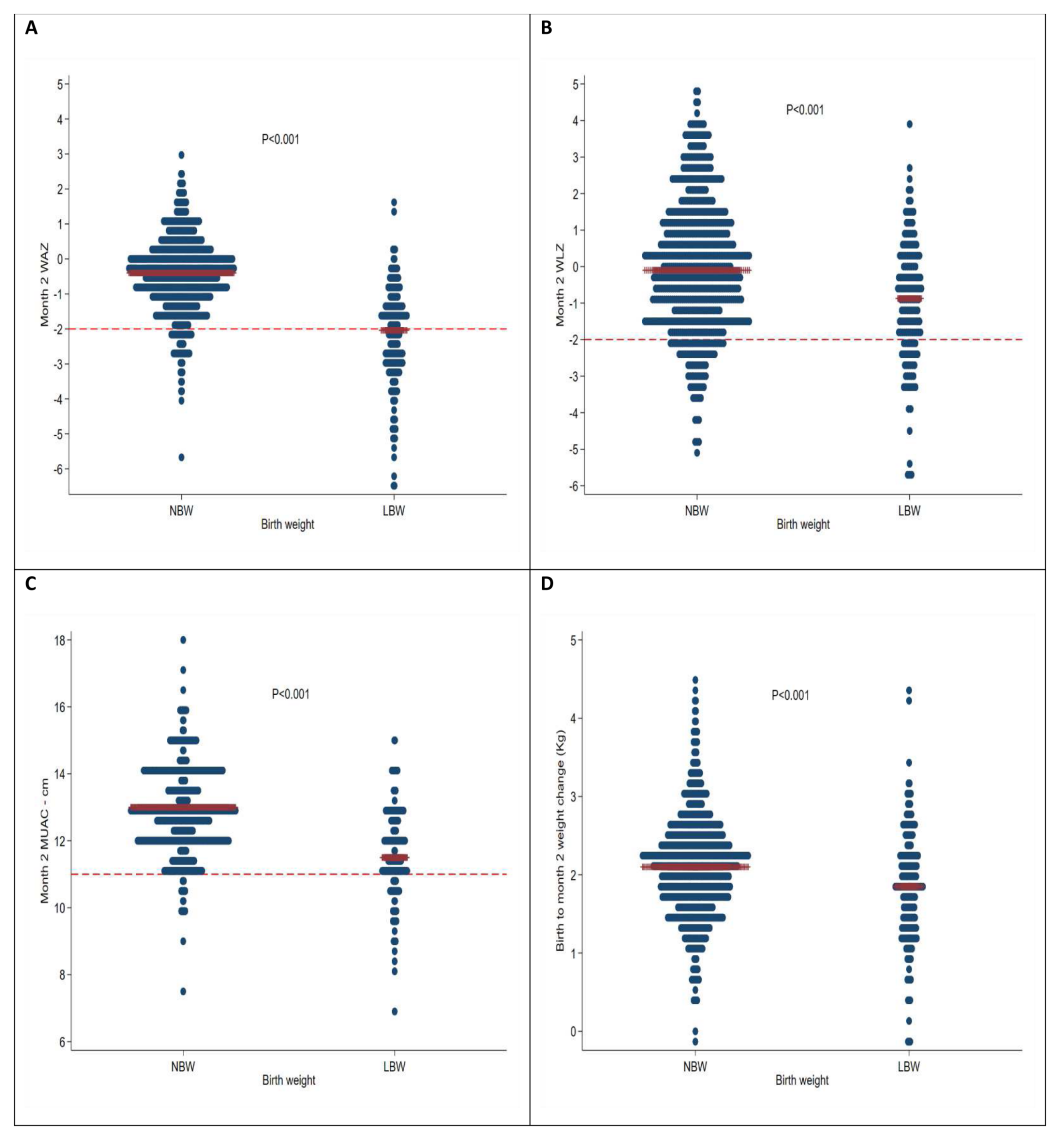
Month two medians of: **A**) WAZ, **B**) WLZ, **C**) MUAC and **D**) birth to month two weight difference. The red bars are the medians, for panel A and B, the dashed line is the cut-off of -2 and panel C MUAC=11 cm.

At two months of age, MUAC had increased by mean (sd) of 2.37 (1.3) cm to 12.3 (sd 1.3); WAZ had increased by 0.27 (sd 1.1) to -0.81 (sd 1.3) Z; and WLZ had increased by mean (sd) of 1.19 (2.2) to -0.19 (2.0) Z; however length-for-age Z score (LAZ) had declined by 0.35 (sd 1.6) to 0.67 (1.7) Z ( Figure 2). The median (IQR) weight increment in Kg from birth to month 2 was 2.2 (1.785 to 2.53) and 1.95 (1.55 to 2.26) for boys and girls respectively which was not significantly different from the WHO increment standards: median (IQR) 2216 (1890 to 2552) grams, P=0.08 for boys and 1897 (1604 to 2210) grams, P=0.13 for girls. The median (IQR) weight (Kg) increment from birth to month 6 was 3.98 (3.4 to 4.5) and 3.6 (3.12 to 4.09) Kg for boys and girls respectively which were significantly lower than the WHO increment standards: median (IQR) 4580 (4072 to 5114) grams P<0.001 for boys and 4079 (3620 to 4597) grams P<0.001 for girls. Anthropometric changes from birth were no better at discriminating mortality compared to single timepoint anthropometric measure taken at month 2, P=0.72, 0.21, 0.28 and 0.80 for WAZ, MUAC, WLZ and LAZ respectively ( Table 3). Results were similar when the regression models of month two measures were adjusted for LBW ( Table 3).

**Table 3.** Comparison in AUCs of single time measurements (month 2) and change between two-time points (birth & month 2).

	Month two measurement	Change between birth & month two	P-value: Changes compared to month two only	Month two measurement adjusted for LBW	P-value: Adjusted compared to unadjusted month two only
	AUCs (95% CI)	AUC (95% CI)		AUCs (95% CI)	
Weight-for-age z-score	0.65 (0.55, 0.74)	0.66 (0.57, 0.75)	0.72	0.66 (0.57, 0.76)	0.44
Mid-upper arm circumference	0.63 (0.53, 0.73)	0.61 (0.51, 0.71)	0.21	0.65 (0.55, 0.76	0.44
Weight-for-length z-score	0.55 (0.44, 0.65)	0.59 (0.50, 0.69)	0.28	0.65 (0.56, 0.75)	0.11
Length-for-age z-score	0.64 (0.54, 0.73)	0.53 (0.55, 0.)	0.80	0.66 (0.56, 0.75)	0.40

P-value from the *roccomp* command comparing the values to month 2.

### LBW and its association with underweight and mortality at two and six months of age

LBW infants were persistently more underweight through the first 12 months of life; the proportion was highest at birth at 82% and lowest at age month 4 at 38% ( Table 2).

**Figure 3.**
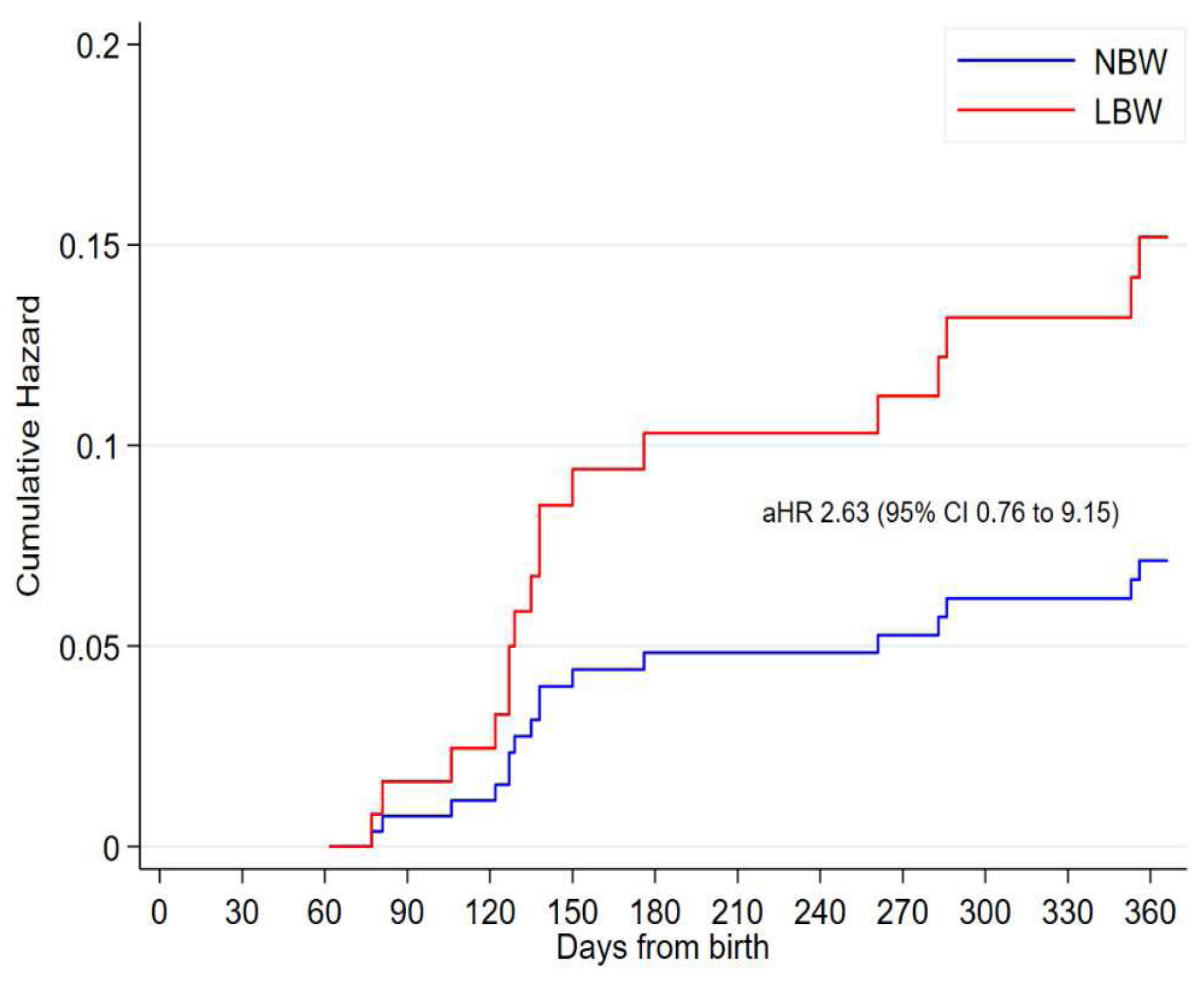
Cumulative hazard of death from two months of age among underweight infants by low birth weight.

At two months, of the 148 (16%) underweight infants, 94 (64%) were LBW ( Table 2). Being underweight was associated with mortality; adjusted hazard ratio (aHR) 1.75 (95% CI 1.04 to 2.79). Among underweight infants at two months, having been born LBW compared to NBW was associated with lower risk of mortality; aHR 2.63 (95% CI 0.76 to 9.15) ( Figure 3).

At six months, 236 (24%) infants were underweight, of whom 92 (49%) had been born LBW ( Table 2). Being underweight was associated with mortality; aHR 2.20 (95% CI 1.06 to 4.55); AUC 0.67 (95% CI 0.57 to 0.77). Among underweight infants at six months, having been born LBW compared to NBW was not associated with lower risk of mortality; aHR 2.43 (95% CI 0.74 to 7.98); AUC 0.66 (95% CI 0.56 to 0.75).

At six months, of the 968 infants assessed, 173 (18%) and 101 (10%) met WHO criteria for moderate acute malnutrition (MAM) and severe acute malnutrition (SAM) respectively. LBW was present in 38/173 (22%) MAM and 39/101 (39%) SAM cases.

## Discussion

We set out to determine if LBW infants who are underweight at month two, which coincides with the age of first vaccination, could be assumed to be growing normally and at a lower risk of mortality than non-LBW underweight infants. We found that being underweight at two or six months of age is associated with a significantly increased risk of mortality irrespective of birth weight. Anthropometric changes observed since birth to two months were no better than a single measure at the point of vaccination in discriminating mortality risk.

LBW was associated with an increased risk of death during the first year of life. This risk is well-documented ^14^. WHO guidelines recommend feeding LBW infants mother’s breastmilk for the first six months of life ^15^, as this is associated with lower incidence of infections and necrotizing enterocolitis than those fed with infant formula ^16^. There is also strong evidence for supplementation with Vitamin D, Iron and Calcium among very low-birth weight (VLBW) infants who are fed on mother’s breastmilk within the first six months of life ^16^. In VLBW and LBW infants, vitamin D supplementation resulted in increases in height, weight, and MUAC in two randomised control trials ^17, 18^. These specific interventions for LBW infants need to be implemented; however, the impact of growth monitoring programmes on growth and mortality of LBW infants is less clear ^15^. A LBW infant tracking below the reference line may be inappropriately considered as “protected” from the risk of mortality because they were born small and apparently growing ‘normally’. Mothers of LBW infants where poor growth is recognised may be advised or initiate supplemental feeds before six months in an attempt to facilitate accelerated growth ^19^, which may actually increase risk. There may also be maternal nutritional and health factors that influence feeding and care, which may usually be missed if the wellbeing of the mother is not assessed with that of her infant. Growth monitoring does present an opportunity if accompanied by informed assessment and appropriate action.******

Emerging evidence suggests that proactive peer support to mothers of LBW infants improves compliance to exclusive breastfeeding in and out of the hospital environment ^20^. Our results indicate that at two months, infants identified to be underweight, irrespective of birth weight, are at increased risk of mortality and should receive targeted support. Underweight infants with a history of LBW should receive micronutrient supplementation as currently recommended, noting the existing gaps in guidance ^16^.

We found that a single MUAC, WAZ or LAZ measure taken at two months of age discriminate mortality risk better than WLZ and that a single measure was better than change from birth. This is an important finding given that WLZ is the currently recommended criterion for intervention and among LBW infants, health worker may consider change in anthropometry more important than the single measure taken at growth monitoring. Our results concur with studies of community infants in The Gambia ^21^ and BukinaFaso ^3^, and from hospital infant cohort in Kenya ^22^ and India ^23^ where among infants u6m, WLZ is not reliably measured ^24, 25^ possibly partly explaining its poor prediction of subsequently mortality. Although using growth velocity may be better at identifying risk, in practice repeated measures may be more complex to implement ^26^. Current evidence suggests that using a MUAC cut-off of <11.0cm and WAZ<-3 when applied at two months (vaccination point) will effectively identify infants with a high risk of subsequent mortality ^21, 22^. As a simple tool, MUAC may be applied at home by either community health workers or family members to help identify at risk infants early ^27^.

In many LMIC settings it is not feasible to distinguish LBW and NBW infants and so there is a need to identify early growth failure in infancy and determine intervention strategies appreciating that LBW infants will comprise a considerable proportion of these. To guide interventions, the Management of At risk Mothers and Infants (MAMI) care pathway approach leverages and connects existing services with active case identification and holistic management of the mother-infant dyad (Emergency Nutrition Network MAMI Tool 2018 available from: https://www.ennonline.net/c-mami).

A strength of this study was the large birth cohort with systematically collected monthly infant anthropometry up to one year of age and their vital status. However, the analysis at month two and six only used data from children with a measured anthropometry. Those excluded because of missing anthropometry could have differed with those included in this analysis. In the absence of ultrasound in pregnancy we were not able to distinguish risks from being preterm versus small for gestational age. A further limitation is that risks and care provision may now differ from those when the data were collected. The data used in this analysis was collected in 2004 and may not reflect improvements in infants care practices in the health system such as improvements in vaccination, community management of acute malnutrition.

## Conclusions

In the first year of life, LBW infants are more likely to be underweight and continue to be at higher risk of mortality than NBW infants. To reduce risk of mortality among infants, research should focus on interventions to prevent LBW and on effective comprehensive interventions to reduce risks of mortality and promote neurodevelopment. Since LBW infants who are underweight have at least the mortality risk of non-LBW infants, all underweight infants need identification during screening or growth monitoring, to have individual nutritional, health and family assessment, and actions to address the risks associated with being underweight.

## Data availability

### Underlying data

Study data were obtained through a data sharing agreement with the School of Public Health, Center of Research in Epidemiology Biostatistics and Clinical Research, Université Libre de Bruxelles, Brussels, Belgium and KEMRI Wellcome Trust Research Programme. The data sharing agreement prohibits sharing of child-level data beyond the research team.

Request to access the de-identified data should be sent to Dr. Paluku Bahwere of School of Public Health, Center of Research in Epidemiology Biostatistics and Clinical Research, Université Libre de Bruxelles, Brussels, Belgium, paluku.bahwere@gmail.com. The request should include: a) full names, designation and affiliation of the person requesting data, b) a copy of study protocol approved by ethics review board showing the methods and statistical analyses plan, and c) proposed period that data will be accessed.
