# Supplementary material for: Growth monitoring and mortality risk in low birthweight infants: a birth cohort study in Burkina Faso
Source: Gates Open Res. 2021 Aug 20;5:82. Originally published 2021 May 13. [Version 2] doi: 10.12688/gatesopenres.13231.2 (PMC10967696; doi:10.12688/gatesopenres.13231.2)
Supplement: Peer review history

# Reviewer report: Reviewer response for version 2

**DOI:** 10.21956/gatesopenres.14603.r35891

**Published:** 2024-03-19

**Authors:** Ekawaty Lutfia Haksari

**Affiliations:** 1 Gadjah Mada University, Yogyakarta, Special Region of Yogyakarta, Indonesia

Please give inclusion and exclusion criteria

 Are there any confounding variables in this study? If yes please give an explanation.

 Discussion: This section contains the author's findings, which are also presented in the Result section and are linked to the study questions. It should be compared with the result of the previous study (or studies) and use the most recent references. Also, it should be explained whether the result supports that of the previous study (or studies) or is in contrast to, or is brand new. The statements should, however, be elaborated and supported by adequate references. In regards to the above matters, the second paragraph should contain the results obtained, and then the results of the previous study/studies are subsequently presented.

Is the work clearly and accurately presented and does it cite the current literature?

Yes

If applicable, is the statistical analysis and its interpretation appropriate?

Yes

Are all the source data underlying the results available to ensure full reproducibility?

Yes

Is the study design appropriate and is the work technically sound?

Yes

Are the conclusions drawn adequately supported by the results?

Yes

Are sufficient details of methods and analysis provided to allow replication by others?

Yes

Reviewer Expertise:

Low birth weight, Small for gestational age infants, Breastfeeding, Kangaroo Mother Care, Neonatal comfort, Audit Maternal Perinatal, Reference Newborn Curve

I confirm that I have read this submission and believe that I have an appropriate level of expertise to confirm that it is of an acceptable scientific standard, however I have significant reservations, as outlined above.

# Reviewer report: Reviewer response for version 2

**DOI:** 10.21956/gatesopenres.14603.r31102

**Published:** 2021-09-03

**Authors:** Ranadip Chowdhury

**Affiliations:** 1 Centre for Health Research and Development, Society for Applied Studies (CHRD-SAS), New Delhi, Delhi, India

The authors have addressed all my comments adequately.

Is the work clearly and accurately presented and does it cite the current literature?

Partly

If applicable, is the statistical analysis and its interpretation appropriate?

Partly

Are all the source data underlying the results available to ensure full reproducibility?

Yes

Is the study design appropriate and is the work technically sound?

Yes

Are the conclusions drawn adequately supported by the results?

Yes

Are sufficient details of methods and analysis provided to allow replication by others?

Yes

Reviewer Expertise:

Drivers of child growth

I confirm that I have read this submission and believe that I have an appropriate level of expertise to confirm that it is of an acceptable scientific standard.

# Reviewer report: Reviewer response for version 1

**DOI:** 10.21956/gatesopenres.14462.r30817

**Published:** 2021-07-13

**Authors:** Elizabeth M. McClure

**Affiliations:** 1 Regional Triangulate Institute International, Research Triangle Park, NC, USA

The investigators conducted a secondary analyses of an old dataset from Burkina Faso to address the question of whether mortality risk differed among underweight LBW infants compared to normal birth weight infants at 2 to 6 months. While this analysis attempts to address an important public health question, there are substantial limitations to the methods, inherent in the older data set (which the authors acknowledge). First, the data is from 2004, and there have been advances in anthropometric measurements since then. Women were only recruited in the 3rd trimester of pregnancy and did not have ultrasound-confirmed GA dating, so we have no information about growth restriction or potential confounders. Also the weight was measured in kg (not g) so there is likely substantial rounding issues and subtle differences may have been lost. Finally, less than half were delivered at a health facility, and the quality of the birth weight for home births is likely to be lower. It would be helpful to include an enrollment flow diagram to help interpret the lost to follow-up at each stage (i.e., from screening through 12 mos follow-up). Overall, while the analytic methods appear to be solid and the research question important, there are substantial limitations with the methods of the original study that reduce the validity of the study findings.

Is the work clearly and accurately presented and does it cite the current literature?

Yes

If applicable, is the statistical analysis and its interpretation appropriate?

Yes

Are all the source data underlying the results available to ensure full reproducibility?

Yes

Is the study design appropriate and is the work technically sound?

Yes

Are the conclusions drawn adequately supported by the results?

Partly

Are sufficient details of methods and analysis provided to allow replication by others?

Yes

Reviewer Expertise:

Perinatal epidemiology

I confirm that I have read this submission and believe that I have an appropriate level of expertise to confirm that it is of an acceptable scientific standard, however I have significant reservations, as outlined above.

# Response

**Published:** 2021-08-18

**Authors:** martha mwangome

1. First, the data is from 2004, and there have been advances in anthropometric measurements since then.

**Response: **We have used 2006 WHO growth standard which are the most updated global reference standards. We have added data on growth increment and compared with 2009 WHO growth increment standards. We feel that it is beyond the scope of the study to include growth velocity analysis as this would introduce a new objective.

 2. Women were only recruited in the 3rd trimester of pregnancy and did not have ultrasound-confirmed GA dating, so we have no information about growth restriction or potential confounders.

**Response:** Yes indeed this is true. Accurate gestational age (GA) dating using ultra sound machines is still, to date, a very difficult and expensive procedure to include in birth cohorts studies in most resource poor settings. In this study in 2004, GA dating was done using last menstrual period (LMP) dating which is a method that is commonly used but inherent to errors. In our analysis, we have used this information and adjusted for being born premature. We have also acknowledged this as a limitation to the study

 3. Also the weight was measured in kg (not g) so there is likely substantial rounding issues and subtle differences may have been lost.

**Response:** The weight measurements were taken to the nearest 10g as recommended and reported to the nearest 1 decimal place (100g). Although the rounding off is likely to have an effect, we think that the effect on the association to the outcome will be very minimal and therefore have decided to leave it as is.

 4. Finally, less than half were delivered at a health facility, and the quality of the birth weight for home births is likely to be lower.

**Response:** For infants delivered in the community, birth weight was measured within the first 48hrs. We think that the timing improves the quality of birth weight as the standard is to measure birth anthropometry within the first 72hrs.

 5. It would be helpful to include an enrollment flow diagram to help interpret the lost to follow-up at each stage (i.e., from screening through 12 mos follow-up).

**Response:** This has now been included. See figure 1.

 6. Overall, while the analytic methods appear to be solid and the research question important, there are substantial limitations with the methods of the original study that reduce the validity of the study findings.

**Response:** It is important to remember that much as an old cohort may come with some limitations (duly acknowledged), we feel that its value far outweighs the limitations. Some of these advantages include;

- It would be impossible to replicate an untreated birth cohort in this day and age and hence impossible to observe nutritional trends as the current ethics restrictions would not allow.
- To date, not many birth cohorts collect MUAC measurement at birth and in the first 6 months of life. The value of MUAC in early infancy is challenging to establish partly because of lack of data. A dataset like this gives an opportunity to explore the potential of expanding the use of MUAC among infants under 6 months.

# Reviewer report: Reviewer response for version 1

**DOI:** 10.21956/gatesopenres.14462.r30680

**Published:** 2021-05-28

**Authors:** Ranadip Chowdhury

**Affiliations:** 1 Centre for Health Research and Development, Society for Applied Studies (CHRD-SAS), New Delhi, Delhi, India

The authors examined data from a birth cohort to compare the discriminatory value for mortality for anthropometry at birth and changes from birth to the following timepoints in infancy: i) at two months of age, which is around the time of infant immunizations; and ii) at six months of age. The authors also investigated overall mortality risk among low birth weight (LBW) infants and whether infants with low anthropometric values measured at two and six months of age. LBW was associated with a lower risk of subsequent mortality. This is an important analysis to understand how to define growth failure in the first six months of life and whether there is a need to have different indicators of LBW infants. Very well conducted analysis and interpretation of the findings. I have very few comments:

1. I would suggest the authors use WHO monthly growth velocity standards to see whether similar findings are obtained.
2. The authors have used the 'gamma frailty model'. Considering the heterogeneity across the sites, I would suggest the authors check the shared frailty assumptions.
3. Do the authors have information on breastfeeding practices, morbidity, care-seeking behaviors, and maternal nutrition status? If not, the authors should mention these in the limitations. If yes, I would suggest presenting this information to better understand the cohort.
4. Why did the authors not use diagnostic accuracies to discriminate the mortality for anthropometry at different time points?
5. I would suggest the authors provide a flow diagram to better understand the loss to follow up at different stages.

Is the work clearly and accurately presented and does it cite the current literature?

Partly

If applicable, is the statistical analysis and its interpretation appropriate?

Partly

Are all the source data underlying the results available to ensure full reproducibility?

Yes

Is the study design appropriate and is the work technically sound?

Yes

Are the conclusions drawn adequately supported by the results?

Yes

Are sufficient details of methods and analysis provided to allow replication by others?

Yes

Reviewer Expertise:

Drivers of child growth

I confirm that I have read this submission and believe that I have an appropriate level of expertise to confirm that it is of an acceptable scientific standard, however I have significant reservations, as outlined above.

# Response

**Published:** 2021-08-18

**Authors:** martha mwangome

1. I would suggest the authors use WHO monthly growth velocity standards to see whether similar findings are obtained.

**Response: **We thank the author for this comment. We however feel that the suggestion to calculate growth velocity and compare these to the WHO growth velocity standards introduces a new objective to the study and is therefore beyond the scope of our current set objectives. However, in keeping with the spirit of the reviewer, we have included the median weight increments from birth to months 2 and 6 months and compared these to the WHO 2009 weights increments standard. We have explained this in the statistical methods and added the findings in the results section (see revised manuscript).

 2. The authors have used the 'gamma frailty model'. Considering the heterogeneity across the sites, I would suggest the authors check the shared frailty assumptions.

**Response: **The underlying assumption when using the 'gamma frailty model' was presence of unobserved heterogeneity across the four recruiting health facilities. We assessed and found evidence of the unobserved heterogeneity using likelihood ratio test in a regression model assessing the effect of LBW on one-year mortality. We have explained this in the statistical method section (see revised manuscript). 

 3. Do the authors have information on breastfeeding practices, morbidity, care-seeking behaviors, and maternal nutrition status? If not, the authors should mention these in the limitations. If yes, I would suggest presenting this information to better understand the cohort.

**Response: **We thank the reviewer for this comment. We confirm that some of these data were collected and we have added on Table 1 and updated paragraph one of the result section to reflect this addition. However, our regression analysis approach was to use a prior confounder, therefore we have not added these variables to the regression analysis.

 4.Why did the authors not use diagnostic accuracies to discriminate the mortality for anthropometry at different time points?

**Response: **We thank the reviewer for this comment. We used receiver operating characteristic (ROC) curve analysis to calculate the area under curve (AUC) for different anthropometry at month 2 and 6 months. We also compared the amount of AUC of discrimination for single time point anthropometry versus difference between two time points (Table 2). The method used to calculate the AUCs and comparison are explained in the statistical methods.

 5. I would suggest the authors provide a flow diagram to better understand the loss to follow up at different stages.

**Response:** Indeed, a flowchart of the participants from birth to month 12, would provide a better understanding of the participants included. We have added a flow chart of the participants as Figure 1.
